# CRISPR/Cas9-mediated homology-directed repair by ssODNs in zebrafish induces complex mutational patterns resulting from genomic integration of repair-template fragments

**DOI:** 10.1242/dmm.035352

**Published:** 2018-10-18

**Authors:** Annekatrien Boel, Hanna De Saffel, Wouter Steyaert, Bert Callewaert, Anne De Paepe, Paul J. Coucke, Andy Willaert

**Affiliations:** Center for Medical Genetics, Department of Biomolecular Medicine, Ghent University, Corneel Heymanslaan 10, 9000 Ghent, Belgium

**Keywords:** CRISPR/Cas9, HDR, Homology-directed repair, Zebrafish, Next-generation sequencing

## Abstract

Targeted genome editing by CRISPR/Cas9 is extremely well fitted to generate gene disruptions, although precise sequence replacement by CRISPR/Cas9-mediated homology-directed repair (HDR) suffers from low efficiency, impeding its use for high-throughput knock-in disease modeling. In this study, we used next-generation sequencing (NGS) analysis to determine the efficiency and reliability of CRISPR/Cas9-mediated HDR using several types of single-stranded oligodeoxynucleotide (ssODN) repair templates for the introduction of disease-relevant point mutations in the zebrafish genome. Our results suggest that HDR rates are strongly determined by repair-template composition, with the most influential factor being homology-arm length. However, we found that repair using ssODNs does not only lead to precise sequence replacement but also induces integration of repair-template fragments at the Cas9 cut site. We observed that error-free repair occurs at a relatively constant rate of 1-4% when using different repair templates, which was sufficient for transmission of point mutations to the F1 generation. On the other hand, erroneous repair mainly accounts for the variability in repair rate between the different repair templates. To further improve error-free HDR rates, elucidating the mechanism behind this erroneous repair is essential. We show that the error-prone nature of ssODN-mediated repair, believed to act via synthesis-dependent strand annealing (SDSA), is most likely due to DNA synthesis errors. In conclusion, caution is warranted when using ssODNs for the generation of knock-in models or for therapeutic applications. We recommend the application of in-depth NGS analysis to examine both the efficiency and error-free nature of HDR events.

This article has an associated First Person interview with the first author of the paper.

## Abstract (precis)

**Summary:** NGS-based analysis reveals that CRISPR/Cas9-induced double-strand-break repair using single-stranded repair templates is error prone in zebrafish, resulting in complex patterns of integrated repair-template fragments.

## INTRODUCTION

Site-specific genome-editing technologies enable the efficient generation of knock-out model systems. However, particularly for disease modeling, there is a growing necessity to complement knock-out models with more precise knock-in models, which will accommodate two needs. First, knock-in models are relevant to study diseases caused by specific and/or recurrent point mutations with dominant-negative, hypomorphic or gain-of-function effects. Second, this approach could serve as an *in vivo* tool to assess the pathogenicity of newly identified variants of unknown significance (VUS), which are massively identified by whole-exome sequencing. CRISPR/Cas9 genome editing is considered the most promising and versatile technology to create knock-in disease models (Cong et al., 2013; Jinek et al., 2012; Mali et al., 2013; Ran et al., 2013a). The CRISPR/Cas9 system induces a double-stranded DNA break (DSB), carried out by the Cas9 nuclease protein, at a specific target site, recognized by the binding of a single-guide RNA (sgRNA) molecule. Following DSB formation, two main endogenous repair mechanisms can be initiated: either the error-prone non-homologous end joining (NHEJ) pathway, often leading to the introduction of indel (insertion/deletion) mutations, or the homology-directed repair (HDR) pathway. HDR is only activated in the presence of a homologous repair template, naturally provided as the sister chromatid during the G2 and S phase of the cell cycle, leading to the reconstitution of the original sequence. The knock-in modeling procedure exploits this mechanism by supplying the CRISPR/Cas9 system with an artificial repair template, homologous to the target sequence and containing base-pair substitutions or gene insertions of interest. Various types of HDR repair templates have been used so far, including both circular and linear double-stranded DNA (dsDNA) molecules, and single-stranded oligodeoxynucleotides (ssODNs) (Salsman and Dellaire, 2017). For large-scale generation of knock-in models, preference is given to the latter as the design and production of ssODNs is easier, less time-consuming and cheaper than the generation of double-stranded templates such as plasmids (Leonetti et al., 2016; Mikuni et al., 2016). Additionally, recently, methods such as ‘easi-CRISPR’ have been developed for the generation of long ssODNs consisting of more than 1 kb of sequence, enabling the insertion of longer sequences such as reporters or gene tags (Miura et al., 2018). Moreover, with the use of ssODNs, illegitimate random integration of the exogenous DNA products into the organism's genome is expected to be less frequent as opposed to the use of dsDNA templates (Won and Dawid, 2017; Würtele et al., 2003; Zorin et al., 2005). Finally, single-stranded templates have shown superiority over similarly designed dsDNA templates in terms of HDR efficiency (Beumer et al., 2013; Miura et al., 2015; Yoshimi et al., 2016).

CRISPR/Cas9-induced HDR-mediated knock-in faces two major problems. First, the mechanism can be error prone. Indeed, imprecise repair events, such as targeted base-pair substitutions combined with insertions and deletions, have been reported (Gratz et al., 2013; Hoshijima et al., 2016; Hwang et al., 2013; Paix et al., 2017; Rivera-Torres et al., 2017; Zu et al., 2013), but the origin and extent of these erroneous repair events has not been thoroughly investigated. Second, its efficiency is relatively low compared to gene disruptions through indel generation. Several cell-endogenous DSB repair mechanisms, including NHEJ and HDR pathways, exist and interact with each other, although the molecular determinants of these interactions are not yet fully known (Kakarougkas and Jeggo, 2014). The decision on which repair pathway is activated is influenced by many factors, including the phase of the cell cycle, DSB complexity, chromatin structure (Brandsma and Gent, 2012; Frank-Vaillant and Marcand, 2002), and the composition and concentration of the repair template (Lin et al., 2014; Rivera-Torres et al., 2017). Previous research explored the impact of adaptations to the length, symmetry and strand complementarity of the ssODN repair template on genome-editing efficiency, although consensus is lacking concerning the impact of these different adaptations (Richardson et al., 2016; Yang et al., 2013). Added chemicals that either block the NHEJ pathway (SCR7, NU7441 and KU0060648) (Ma et al., 2016; Maruyama et al., 2015; Singh et al., 2015; Srivastava et al., 2012) or stimulate the HDR pathway (RS-1, L755507) (Jayathilaka et al., 2008; Pinder et al., 2015; Song et al., 2016; Yu et al., 2015) improve HDR rates in cellular systems to a certain extent, but still need further evaluation in other models.

The zebrafish is frequently used for disease modeling because of its many advantages, such as a rapid development, large offspring numbers, and the ease and speed in generating mutant lines. In this study, we evaluated the efficiency of point-mutation knock-in approaches in zebrafish models. We evaluated the effect of length, symmetry and strand complementarity of ssODN repair templates on HDR-mediated knock-in rates at multiple sgRNA target sites, and assessed a further impact of multiple chemical compounds that either block NHEJ or stimulate HDR. With next-generation sequencing (NGS), we accurately determined precise and erroneous knock-in efficiencies. Our work provides new insights into the effect of repair-template composition on HDR efficiency and stresses the importance of rigorous analysis of CRISPR/Cas9-edited genomic targets to evaluate the error-free nature of the obtained knock-in.

## RESULTS

### HDR is prone to errors and influenced by ssODN repair-template composition

**Fig. 1.**
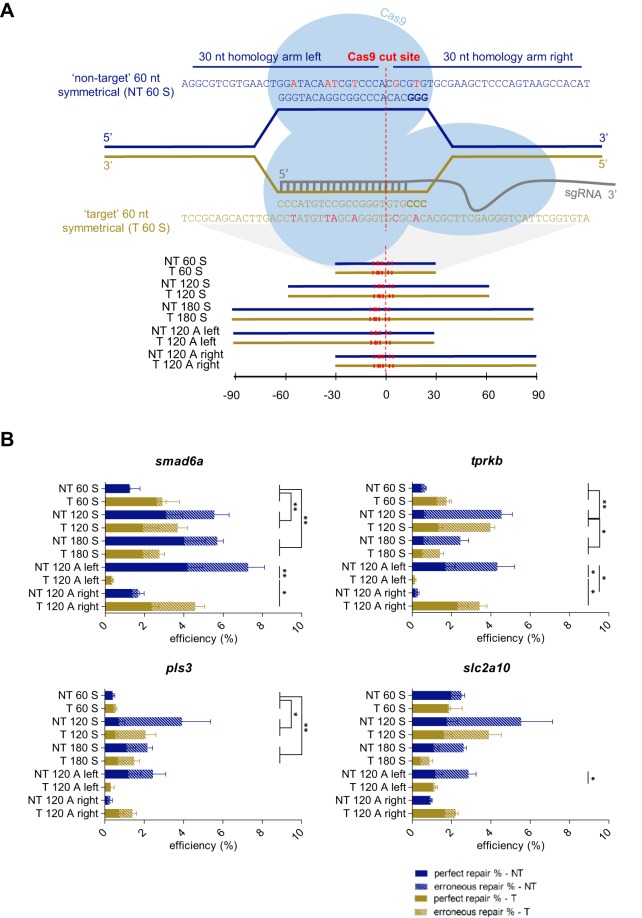
**Impact of repair-template homology-arm length, strand complementarity and symmetry on HDR efficiency.** (A) Illustration of an sgRNA target site for the zebrafish *smad6a* gene, with protospacer sequence GGGTACAGGCGGCCCACAC. Following Cas9 recruitment and sgRNA binding, the DNA is cleaved 3 bp upstream of the PAM sequence (NGG, displayed in bold), which is visualized by the dashed red line and indicated as the ‘Cas9 cut site’. We designed ten repair templates, 60, 120 or 180 nucleotides (nt) in length, either corresponding to the sgRNA target strand (‘target’ – T) or to the complementary strand (‘non-target’ – NT). The repair templates were either symmetrically (S) or asymmetrically (A) positioned around the Cas9 cut site. The position of the different repair templates relative to the Cas9 cut site is depicted in the lower panel. The sequence of the 60 nt repair templates for *smad6a* is shown in the upper panel, and the nucleotide sequences of all other repair templates are listed in Table S8. Each repair template contains several synonymous nucleotide changes, depicted in red, relative to the reference sequence, including replacement of a guanine nucleotide in the PAM, whenever possible. (B) For each target site (*smad6a*, *tprkb*, *pls3* or *slc2a10*) and each repair-template type (NT 60 S, NT 120 S, NT 180 S, NT 120 A left, NT 120 A right, T 60 S, T 120 S, T 180 S, T 120 A left, T 120 A right), average total HDR efficiencies resulting from five independent experiments were plotted. Calculated repair rates represent the number of sequencing reads containing the intended base-pair substitution closest to the Cas9 cut site. The HDR rates were split up into two categories: ‘perfect repair %’, representing the percentage of NGS reads containing at least the base-pair change closest to the Cas9 cut site (plain bars), and ‘erroneous repair %’, representing the reads containing erroneous integration events of repair-template fragments (dashed bars). NT, non-target; T, target; 60-120-180, total repair-template length; S, symmetrical; A, asymmetrical. Error bars represent the s.e.m. for five independent biological replicates each consisting of a pooled sample of 20 embryos. Repair rates depicted in this graph are listed in Table S1. Statistical tests performed: one-way ANOVA with blocking (60 nt vs 120 nt, 60 nt vs 180 nt, 120 nt vs 180 nt, target vs non-target) for symmetrical templates, non-parametric Kruskal–Wallis test, followed by pairwise comparison with Dunn–Bonferroni correction, for asymmetrical templates; *P<0.05 and **P<0.01.

A prerequisite for activation of the HDR pathway is the presence of a suitable repair template. For each of four different sgRNA target sites in the zebrafish genome (located in the *smad6a*, *tprkb*, *pls3* and *slc2a10* genes), we evaluated multiple ssODN repair-template compositions, altering length, strand complementarity and symmetry, in order to maximize HDR efficiency (Fig. 1A). The results reveal a major impact of repair-template composition on HDR efficiency. In three out of four sgRNA targets, the HDR rate for symmetrical templates increased significantly when extending the template length from 60 to 120 nucleotides (nt), with the maximum improvement being tenfold. Lengthening the template further to 180 nt moderately decreased HDR rates in most cases (Fig. 1B, Table S1). Overall, we obtained maximal total HDR rates ranging from 4 to 8%, depending on the target site. Strand complementarity did not significantly affect HDR rates, although templates that share sequence identity with the strand that is not binding the sgRNA (‘non-target’ templates) tend to perform slightly better. Asymmetrically designed templates did not result in better HDR rates compared to symmetrically designed templates: two asymmetrical repair templates, a ‘non-target’ template with its long homology arm at the protospacer adjacent motif (PAM)-distal side (‘NT 120 A left’) and a ‘target’ template with its long homology arm at the PAM-containing side (‘T 120 A right’), performed at similar levels compared to the 120 nt symmetrical templates; however, their counterparts with different strand complementarities (‘NT 120 A right’ and ‘T 120 A left’) performed remarkably worse and resulted in the lowest repair rates observed.

**Fig. 2.**
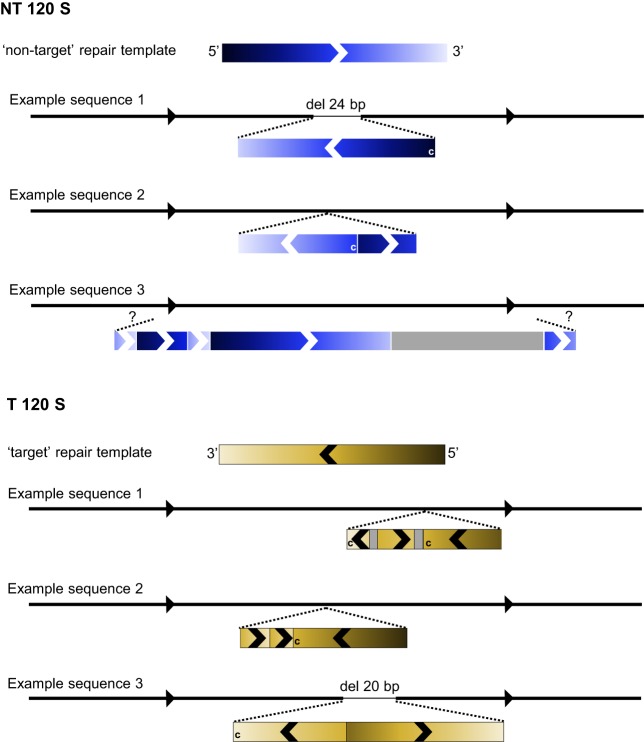
**Simplified schematic representation of erroneous repair-template integration events at the *smad6a* zebrafish gene.** For both the ‘NT 120 S’ and ‘T 120 S’ repair templates, three examples of NGS reads are schematized to clarify the erroneous repair patterns that were encountered in this study. For each example, the black line with arrows represents the reference sequence, and the two black dashed lines indicate the approximate location of repair-template insertions. Inserted fragments are depicted as rectangles, colored in blue or yellow, in accordance with the used repair template (indicated in the scheme as ‘non-target’ or ‘target’ repair template on top of the three example sequences). Gray rectangles depict ‘random’ sequences not corresponding to the repair-template sequence. The color gradient clarifies to which part of the repair template the insertional fragment corresponds and defines the orientation of the fragment. This is additionally illustrated by arrows overlaying the rectangles. A ‘c’ in the rectangle means that it corresponds with the repair template's complementary sequence. In ‘NT 120 S’ example sequence 3, the integrated repair template fragments could not be assigned to a certain location in the reference sequence, due to the limited length of NGS sequence reads (250 bp), which is depicted by two question marks ('?'). This scheme is a simplified representation. A more detailed scheme of these examples, using the NGS read sequence as reference point, is depicted in Fig. S2.

Further dissection of the nature of the repair events using in-depth NGS data analysis revealed that ssODN-mediated DSB repair does not only lead to scarless genome editing with introduction of the intended base pair change(s), but unexpectedly also causes erroneous integration of ssODN repair-template fragments at the site of the DSB. Consequently, HDR rates dropped from 4-8% (total) to 1-4% (perfect) when removing all NGS reads containing erroneous ssODN integration events from the analysis (Fig. S1). These reads could only be partially aligned to the reference genome. The parts of the reads that were clipped contained fragments of the targeted gene sequence, indicating multiple and often partial erroneous integrations of the repair template. We schematized a number of erroneous integration events of the *smad6a* ‘NT 120 S’ and ‘T 120 S’ repair templates to illustrate the wide diversity and complexity of integration patterns (Fig. 2, Fig. S2, Table S2). These patterns ranged from an inverse integration of a part of the repair template to the introduction of multiple repair-template fragments that can be present in either orientation. Additionally, in some cases, a partial deletion of the sequence at the targeted locus was observed.

Remarkably, the observed differences in total HDR rates between the different symmetrical repair templates can be largely attributed to the difference in frequency of erroneous integration events (Fig. 1B, Fig. S1, Table S1). In contrast, the difference in HDR rates between the asymmetrical repair templates did not change when removing all NGS reads containing erroneous ssODN integration events; ‘NT 120 A left’ and ‘T 120 A right’ still outperformed their counterparts with different strand complementarities, when only considering perfect repair rates. In addition, for three out of the four sgRNA target sites, perfect repair rates for these ‘superior’ asymmetrical templates (‘NT 120 A left’ and ‘T 120 A right’) are higher than for their symmetrical counterparts (‘NT 120 S’ and ‘T 120 S’). Nevertheless, no specific type of repair template performed consistently best across all four sgRNA target sites.

### Inverse relation between distance of base-pair substitution relative to Cas9 cut site and knock-in efficiency

**Fig. 3.**
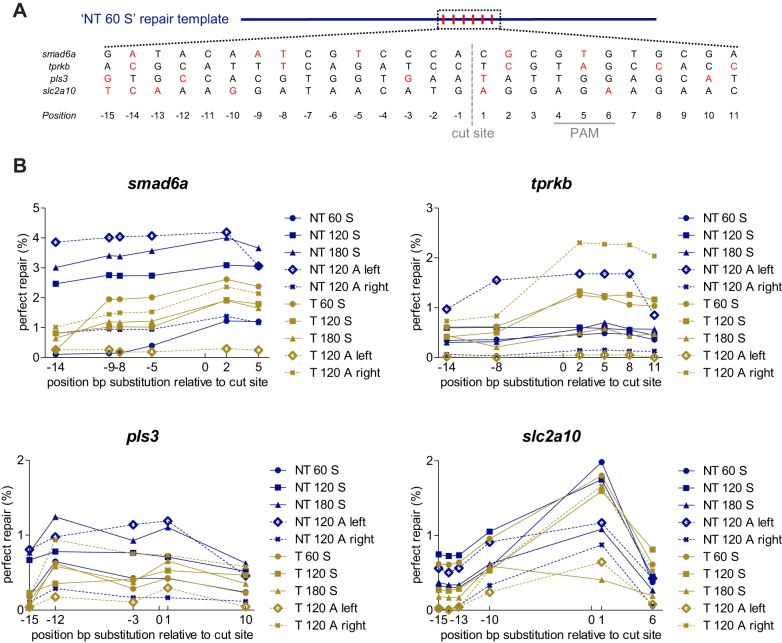
**Impact of the position of the substituted nucleotide in the repair template on repair rates.** (A) Repair-template composition with specified locations of the base pair alterations, shown for an ‘NT 60 S’ repair template. Each repair template included in this study contains several synonymous nucleotide changes relative to the reference sequence. Whenever possible, a guanine nucleotide of the PAM was replaced, in order to avoid undesired cleavage of the repair template by Cas9. In addition, five nucleotide changes were included in the region surrounding the Cas9 cut site (designated as point zero, visualized with a gray dashed line). All nucleotide changes are depicted in red. The position of the PAM sequence is marked with a full gray line. (B) The HDR efficiency (perfect repair only) for each base-pair substitution in each repair template is plotted as a function of the relative distance to the Cas9 cut site. Each plotted data point shows average values of five independent experiments, each analyzing 20 embryos. To improve clarity, error bars were omitted. NT, non-target; T, target; 60-120-180, total repair-template length; S, symmetrical; A, asymmetrical. Repair rates depicted in this graph are listed in Table S3.

The presence of multiple synonymous nucleotide substitutions in the repair templates (Fig. 3A) allowed us to investigate the impact of the position of the substituted base pair relative to the Cas9 cut site on perfect repair rates (Fig. 3B, Table S3). Successful introduction of the substitution was generally highest at the positions close to the Cas9 cut site, and slightly dropped proportionally with increasing distance. The extent of this drop in HDR rates varied for the different sgRNA target sites. Comparing the incorporation rate of the nucleotide alteration at the position furthest (−15 or −14) and closest (1 or 2) from the Cas9 cut site, averaged for all repair templates of a specific gene, revealed a decrease in perfect repair rates that is twofold for *tprkb*, fourfold for *pls3* and *smad6a*, and 24-fold for *slc2a10*. The biggest reduction in nucleotide alteration rates at the edge positions was noted for some of the repair templates that contain short homology arms (‘T 60 S’, ‘NT 60 S’ and the asymmetrical templates).

### Chemical compound administration does not influence HDR rates in zebrafish

**Fig. 4.**
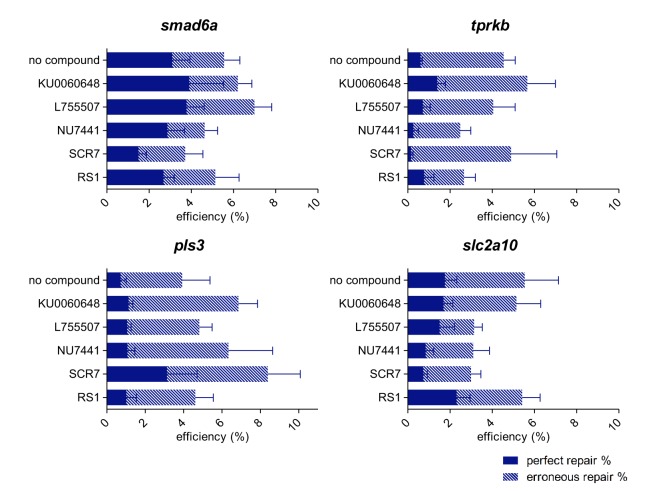
**Influence of chemical compound administration through injection on HDR efficiency.** Injection mixes containing the NT 120 S repair template were complemented with chemical compounds that either inhibit specific components of the NHEJ pathway, including SCR7, NU7441 and KU0060648, or that were shown to stimulate the HDR pathway, including RS1 and L755507. Five independent experiments were carried out and average total HDR rates are shown, split into two categories: perfect repair % (plain bars) and erroneous repair % (dashed bars). Error bars represent the s.e.m. for five independent biological replicates each consisting of a pooled sample of 20 embryos. Repair rates depicted in this graph are listed in Table S4. Statistical tests performed: independent samples *t*-test for normal distributed groups and the non-parametric independent samples Mann–Whitney *U*-test for non-normal distributed groups.

We aimed to increase HDR rates in zebrafish by the use of chemical compounds that, in cell cultures, were shown to either block the NHEJ pathway (SCR7, NU7441, KU0060648) or stimulate the HDR pathway (RS1, L755507). We either co-injected these compounds with Cas9/sgRNA complexes targeting one of the four genes together with a corresponding symmetrical 120 nt repair template (‘NT 120 S’) or incubated injected embryos for 24 h in compound screening medium, containing the chemical compounds. For injection, the administered compound dose depended either on compound solubility [selection of the highest compound concentration without aggregate formation in the injection mix (L755507)], compound toxicity [selection of the highest compound concentration without any influence on embryo development and survival (SCR7 and RS1)] and practical considerations [use of the maximal free volume in the injection mix used for compound administration (NU7441 and KU0060648)]. For incubation, the maximal compound concentration for which more than 80% of the embryos developed normally was selected. None of the administered compounds had a notable influence on HDR rates, neither for compound injection (Fig. 4, Table S4) nor for compound incubation (Fig. S3, Table S5).

### Precise base-pair substitutions introduced via ssODNs show germline transmission

**Fig. 5.**
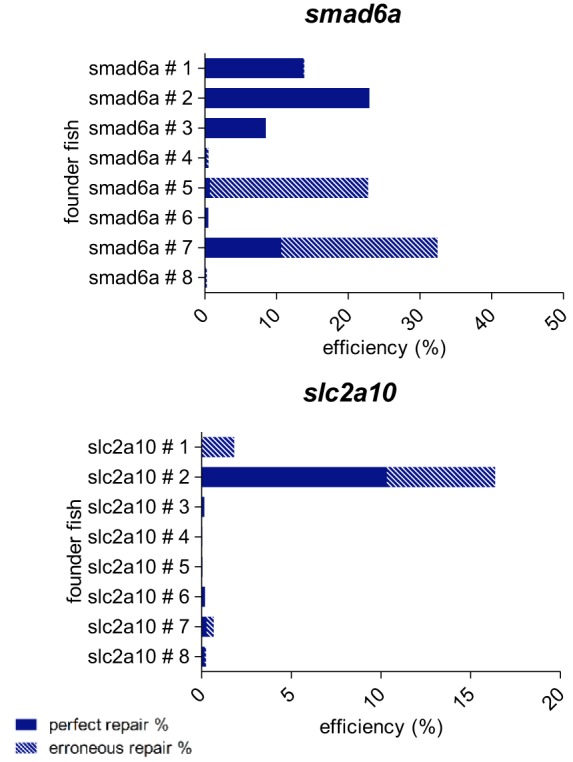
**Determination of germline transmission of precise base-pair substitutions introduced by CRISPR/Cas9-mediated HDR.** For two of the four target sites included in this study (the *slc2a10* and *smad6a* zebrafish genes), embryos injected with injection mixes containing the NT 120 S repair template were grown until adulthood. For each target site, eight adult fish (labeled founder fish # 1, # 2, … # 8) were screened for the presence of the precise base-pair substitutions located closest to the Cas9 cut site in their germ cells. Therefore, DNA was extracted from collected eggs or sperm, and subjected to NGS analysis. HDR rates are shown, split into two categories: perfect repair % (plain bars) and erroneous repair % (dashed bars). Repair rates depicted in this graph are listed in Table S6.

We investigated the efficacy of germline transmission of base-pair substitutions introduced via HDR-mediated knock-in using ssODN repair templates. At the age of 3 months, we collected germ cells from eight fish per target (*smad6a* and *slc2a10*) and performed NGS. Analysis of the nucleotide alteration located closest to the Cas9 cut site revealed a relevant number of germ cells with HDR events in, respectively, five out of eight and two out of eight screened fish (Fig. 5, Table S6). Most of these founder fish contained germ cells with both perfect repair and erroneous events but, for both gene targets, we identified at least one founder fish with scarless HDR rates above 10%. This provides a good chance of transmission of the nucleotide alteration to the next generation, despite the relatively low perfect repair rates detected during sequence analysis of injected embryos at 1 dpf.

## DISCUSSION

The generation of knock-in models has been greatly simplified by the emergence of CRISPR/Cas9-mediated HDR approaches, where genomic target sequences are replaced by homologous donor repair templates. Nevertheless, the efficiency of this process is low, precluding its use for high-throughput disease modeling or therapeutic applications. This study evaluates different approaches to improve the efficiency of scarless CRISPR/Cas9-mediated knock-in of single-base-pair substitutions by using ssODN repair templates in zebrafish, and accurately assesses total, ‘erroneous’ and ‘perfect’ repair rates by deep-sequencing analysis.

In a first step, we evaluated the most effective composition of the ssODN repair template for four different sgRNA target sites in the zebrafish genome. Repair-template length significantly affects total repair rates, with 120 nt being identified as the most optimal length to promote HDR. Shorter templates performed significantly worse, while longer templates showed a moderate drop in HDR rates. These findings are comparable to results obtained in human stem cells (Yang et al., 2013), pigs (Wang et al., 2016) and mice (Shen et al., 2013), where reported optimal total lengths approximate 90, 120 and 100 nt, respectively. We obtained average total HDR-mediated knock-in rates of 4-8% depending on the sgRNA target site when using 120 nt templates. This is more or less comparable to the results of Hruscha et al. (2013), to our knowledge the only other study in zebrafish evaluating HDR using NGS on whole-embryo DNA extracts. Other studies that investigated nuclease-mediated HDR in zebrafish only report on percentages of embryos containing at least one precise editing event, or make estimations on HDR efficiency based on PCR- or restriction-assay-derived data, impeding a direct comparison with our results (Albadri et al., 2017). The inclusion of repair templates that are complementary to either the sgRNA target or non-target strand enabled us to investigate the impact of strand preference on HDR rates. Our data do not support a clear strand preference for symmetrical repair templates at four different sgRNA target sites. This is in agreement with data obtained in other organisms (Lin et al., 2014; Renaud et al., 2016), but does not rule out specific locus-dependent strand preference as suggested previously (Hwang et al., 2013).

**Fig. 6.**
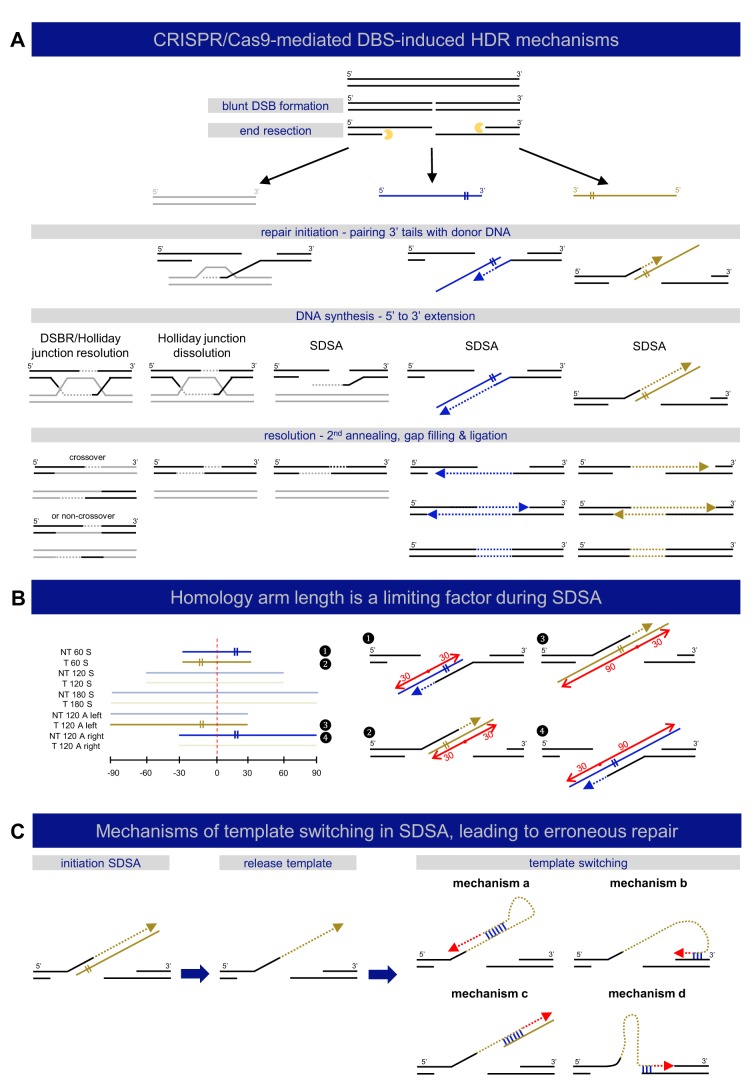
**Mechanisms of DNA double-strand break repair.** (A) A schematic representation of different described mechanisms of DSB-induced HDR is shown, categorized by repair template type: double-stranded (gray) or single-stranded (‘target’ – yellow, ‘non-target’ – blue). DSB repair by HDR is always initiated by DNA end resection, resulting in 3′ single-stranded DNA tails, which participate in strand invasion into homologous sequences. DSB repair with double-stranded repair templates can be carried out by three different mechanisms: double-strand break repair (DSBR or Holliday junction resolution), Holliday junction dissolution, or synthesis-dependent strand annealing (SDSA) (Allers and Lichten, 2001; Szostak et al., 1983). DSB repair using single-stranded templates, sometimes referred to as SSTR, acts via SDSA (Davis and Maizels, 2016; Kan et al., 2017; Paix et al., 2017). (B) Homology-arm length influences the efficiency of SDSA. The left panel depicts the composition of all included repair templates in this study, where the four templates that perform significantly worse in comparison to the others in terms of total repair rates (see Fig. 1B) are highlighted (1-4). The location of the DSB is marked with a red dashed line. These four templates all contain a short (30 nt) homology arm at the side of the repair template that does not anneal to one of 3′ overhangs (marked with ‘II’ sign), but participates in the resolution step, as also depicted in the right panel, where each of these inefficient templates is fitted to the SDSA model for DSB repair with single-stranded templates. The red dots refer to the DSB location, with left and right homology arms marked with red arrows and length indications. (C) Mechanisms of template switching in SDSA leading to erroneous repair. Following SDSA initiation, dissociation of unstable intermediates can lead to template switching. Four possible mechanisms for template switching during SDSA are shown. These mechanisms are based on microhomology (binding marked in blue) and can result in erroneous replication (red dashed arrows).

Next, we examined the impact of repair-template symmetry on HDR rates. A recent study has suggested that use of asymmetrical templates leads to improved HDR rates; Richardson et al. (2016) showed that, in cell cultures, complete dissociation of the Cas9-DNA complex after DSB creation is preceded by an early release of the PAM-distal non-target strand (Richardson et al., 2016). This insight might contribute to rational repair-template design, particularly by constructing asymmetrical templates complementary to the non-target strand, and with homology arms of 90 nt and 30 nt, corresponding respectively to the PAM-containing and the PAM-distal side of the DSB. Exploration of the performance of this specific repair template in zebrafish, alongside a number of other asymmetrical templates complementary to either the ‘target’ or ‘non-target’ strand, did not confirm that the abovementioned optimal asymmetrical repair template (designated ‘T 120 A right’ in our study), or any other asymmetrical template, performed notably better than the symmetrical repair templates. Interestingly, though, significant differences within the set of asymmetrical repair templates were noticeable, with ‘NT 120 A left’ and ‘T 120 A right’ clearly outperforming the other two asymmetrical repair templates. This remarkable difference, together with our finding that short 60 nt templates perform significantly worse than 120 nt or 180 nt templates, adds to our understanding of the mechanisms involved in DSB repair. A currently emerging hypothesis suggests that DSB repair using single-stranded templates proceeds through the single-stranded template repair (SSTR) pathway (Jasin and Haber, 2016). This process occurs independently of RAD51 and BRCA2, the key components of homologous recombination (HR) (Bothmer et al., 2017). It is proposed that the mechanism behind SSTR is based on synthesis-dependent strand annealing (SDSA), consisting of three major steps: (1) resection of the DSB to create 3′ overhangs, (2) pairing of the overhangs with the donor DNA followed by extension of the 3′ strand by DNA-polymerase-mediated DNA synthesis (initiation), and (3) strand displacement of the extended 3′ strand from the template strand with reannealing of the extended 3′ strand and the endogenous DNA at the opposite end of the DSB (resolution) (Fig. 6A). This process is characterized by unidirectional sequence conversion (Davis and Maizels, 2016; Kan et al., 2017; Paix et al., 2017). Templates containing a short (30 nt) homology arm at the side of the repair template that does not anneal to one of 3′ overhangs (‘NT 60 S’, ‘T 60 S’, ‘T 120 A left’, ‘NT 120 A right’), but participates in the resolution step, perform poorly in our study (Fig. 6B). These findings point towards a necessity for these particular homology arms to have an optimal length (about 60 nt) for resolution, at least in the zebrafish. The difference in performance between the asymmetrical templates included in this study may thus be linked to SDSA pathway requirements, instead of being attributed to the nature of the dissociation of the Cas9-DNA complex after DSB, as suggested by Richardson et al. (2016).

The outcome of precise genome editing using ssODNs depends on the perfect replacement of the target sequence by the repair template. Extended data analysis of the obtained deep-sequencing results in this study revealed that approximately half of the sequencing reads containing the base-pair alterations of interest additionally show a complex pattern of multiple integrated fragments of the repair template that can be present in both directions. This finding was not entirely unexpected, since a number of studies have reported imprecise HDR edits (Gratz et al., 2013; Hwang et al., 2013; Paix et al., 2017; Rivera-Torres et al., 2017; Zu et al., 2013), but the extent of this phenomenon was unanticipated. It is generally believed that the use of ssODNs, unlike dsDNA templates, circumvent random and illegitimate integration of the exogenous DNA products into the organism's genome (Won and Dawid, 2017; Würtele et al., 2003; Zorin et al., 2005). Therefore, the erroneous integration events encountered in this study are most likely attributed to mechanisms other than random integration. Since ssODN-induced DSB repair most likely occurs through SDSA (Paix et al., 2017), initiating DNA replication steps may be prone to polymerase slippage and stalling (Rodgers and McVey, 2016) with subsequent dissociation of unstable intermediates and template switching (Rodgers and McVey, 2016). Multiple rounds of template switching can lead to complex mutational spectra (Guirouilh-Barbat et al., 2014). Correspondingly, template switching during CRISPR/Cas9-mediated HDR in HEK293T cells has been recently demonstrated by Paix et al. (2017). We hypothesized that the presence of microhomologies in the target sequence may explain why certain targets are prone to template switching with subsequent erroneous repair, as previously seen in genomic rearrangement events (Löytynoja and Goldman, 2017) (Fig. 6C). As a proof-of-principle, we showed, for one of the repair templates targeting the *smad6* gene, that erroneous repair occurred through multiple template switching events during SDSA-based DSB repair, based on the presence of short microhomology sequences (Fig. S4). The sequence-specific nature of these events holds promise for future development of prediction algorithms for erroneous repair in CRISPR/Cas9-mediated HDR.

Importantly, no repair template performs unambiguously best across all sgRNA target sites, when solely considering perfect repair rates, although there is a tendency for some asymmetrical repair templates (‘NT 120 A left’ and ‘T 120 A right’) to outperform their symmetrical counterparts (‘NT 120 S’ and ‘T 120 S’), comparable to previously reported results in zebrafish (Prykhozhij et al., 2018). Therefore, it is advisable to try different types of repair templates when attempting to knock-in a point mutation of interest.

Our data clearly indicate that the outperformance of some of the templates when considering total HDR rates was mainly due to erroneous repair. Excluding the sequencing reads that contain erroneous integration events leads to a drop in HDR rates (of the base pair located closest to the DSB site) from 4-8% to 1-4%, depending on the sgRNA target site. Moreover, these perfect repair rates drop even further if the distance between the base alteration and the Cas9 cut site increases, a phenomenon that was observed previously (Elliott et al., 1998; Paquet et al., 2016; Ran et al., 2013b). This implies that the distance between the Cas9-induced DSB and the intended sequence alteration should be minimized. Despite the low rates of scarless repair in zebrafish embryos, efficient transmission of nucleotide alterations to subsequent generations was found to be relatively efficient.

Finally, we evaluated HDR rates following chemical intervention blocking the NHEJ pathway (SCR7, NU7441, KU0060648) or stimulating the HDR pathway (RS1, L755507), an approach reportedly successful in cellular systems. Two delivery methods (incubation and injection) were applied. Surprisingly, none of these compounds affected either perfect or erroneous HDR rates in zebrafish. Possible explanations include: (1) delivery through injection or compound toxicity in the zebrafish hampers the delivery of the minimal compound concentration necessary for its intended activity, (2) the absence of a response to compound administration could be attributed to species differences, although it is known that DSB repair mechanisms are generally well conserved, or (3) the RAD51 stimulator RS1 might only have an effect when using double-stranded repair templates, since single-stranded template repair of DSBs is believed to act independently from RAD51 (Bothmer et al., 2017). In view of the latter, compounds that induce the SSTR pathway could have better potential in increasing HDR rates when using single-stranded templates. Interestingly, a recent study in zebrafish has shown that SCR7 administration through incubation increased HDR rates from 5 to 13% using an ssODN donor (Zhang et al., 2018). One possible reason that our results conflict with their conclusion is the difference in analysis method. We report repair rates that match the actual frequency of altered alleles in a pool of embryos, determined by NGS, while the study of Zhang and co-workers only reports the percentage of embryos containing HDR events. Moreover, these HDR rates are determined by a method solely based on PCR amplification. The use of PCR-based techniques using primers that specifically recognize the mutant sequence introduced by HDR should be avoided since they cannot distinguish erroneous repair from perfect repair and may thus lead to an overestimation of error-free HDR events. In addition, this approach lacks sensitivity to detect small differences in HDR rates. Another study performed in *Xenopus* showed that, similarly to our study, SCR7 was toxic to embryos and did not improve HDR rates (Aslan et al., 2017). Administration to non-dividing oocytes, on the other hand, appeared to significantly increase HDR rates. Therefore, the procedure of oocyte treatment could be a challenging but promising approach for future experiments in zebrafish, aiming to increase HDR rates (Xie et al., 2016).

Overall, in this study we tested the performance of multiple strategies in the zebrafish model system that were previously shown to improve HDR rates in cellular systems, including optimal donor template design and chemical compound administration. We demonstrate that HDR-mediated knock-in is influenced by the design of the ssODN template, while none of the investigated chemical compounds significantly increased HDR rates in zebrafish. Importantly, we identified that imprecise complex genomic insertions of the donor template constitute a significant portion of total HDR events, and suggest that repeated template switching during SDSA is the underlying mechanism of erroneous ssODN-mediated HDR. These findings stress the need for optimal experimental design and reliable assessment of HDR-mediated knock-in in zebrafish (Box 1).

**Box 1. Conclusions and guidelines for design and analysis of CRISPR/Cas9-mediated HDR experiments in zebrafish**

**Experimental setup**

• None of the repair templates included in this study performs unambiguously best in terms of scarless HDR rates (perfect repair).

• Repair-template design determines the extent of erroneous integration of repair-template fragments (erroneous repair).

• Repair templates with different strand complementarities (‘target’ vs ‘non-target’) perform similarly.

• Specific types of 120 nt asymmetrical repair templates (designated ‘NT 120 A left’ and ‘T 120 A right’ in this study) have a tendency to outperform 120 nt symmetrical repair templates.

• The distance between the Cas9 cut site and the base-pair substitution should be minimized.

• Chemical compound administration (SCR7, NU7441, KU0060648, RS1, L755507), both through injection or by incubation, does not increase HDR rates in zebrafish embryos

**HDR analysis**

• Methods solely based on PCR amplification (for example with one primer targeting the modified sequence) or restriction digests are not suitable to detect erroneous HDR.

• Next-generation sequencing (NGS) or Sanger sequencing of a region substantially larger than the repair-template length is advised to be able to detect erroneous repair.

## MATERIALS AND METHODS

### sgRNA design and production

We designed sgRNA molecules targeting four different zebrafish genes (*smad6a*, *tprkb*, *pls3* and *slc2a10*) with CRISPRdirect software (http://crispr.dbcls.jp/) (Naito et al., 2015). For *in vitro* transcription, we synthetized gBlocks with the following structure (IDT, gBlock): 5′-CCGCTAGCTAATACGACTCACTATA-**GG**-**N_18_**-GTTTTAGAGCTAGAAATAGCAAGTTAAAATAAGGCTAGTCCGTTATCAACTTGAAAAAGTGGCACCGAGTCGGTGCTTTT-3′, where the two guanines in bold fulfill the T7 polymerase promoter requirement and N_18_ represents the last 18 nucleotides of the protospacer sequence. Specific gBlock sequences for the four targets are listed in Table S7. Lyophilized gBlock molecules were dissolved in nuclease-free water at a concentration of 10 ng/µl and 4 µl was used as an input for *in vitro* transcription, using the MEGAshortscript™ T7 Transcription Kit (Invitrogen, cat. no. AM1354). We followed the general guidelines of the manufacturer except for the duration of the incubation step at 37°C, which was carried out overnight to obtain a maximum yield. The transcription reaction was purified using the MEGAclear™ Kit (Life Technologies, cat. no. AM1908), following the manufacturer's instructions. Quantity and integrity of the RNA was determined using, respectively, the DropSense96 device (Trinean) and the Experion microfluidic capillary electrophoresis system (Bio-Rad). The RNA was aliquoted and stored at −80°C.

### Repair-template design

For each of the four different sgRNA target sites (located in the *smad6a*, *tprkb*, *pls3* and *slc2a10* genes), we designed ten ssODN repair templates (Fig. 1A). Six templates contained symmetrical homology arms, flanking the theoretical Cas9 cut site (located 3 base pairs upstream of the PAM), with total lengths of 60 nt, 120 nt or 180 nt and sharing sequence identity either with the sgRNA-binding strand (often referred to as ‘antisense’, here designated as ‘target’: ‘T 60 S’, ‘T 120 S’, ‘T 180 S’), or with the complementary strand (often referred to as ‘sense’, here designated as ‘non-target’: ‘NT 60 S’, ‘T 120 S’, ‘T 180 S’). Four asymmetrical repair templates consisted of 30 nt and 90 nt homology arms, corresponding, respectively, to the PAM-distal and PAM-proximal side relative to the theoretical Cas9 cut site or *vice versa* and sharing sequence identity with either the sgRNA target (‘T 120 A right’ and ‘T 120 A left’) or non-target (‘NT 120 A right’ and ‘NT A 120 left’) strand. Whenever possible, we masked the sequence corresponding to the PAM by replacing a guanine with another nucleotide, introducing a synonymous mutation. Each of the ten repair templates further contained five nucleotide substitutions in a region from 15 nt upstream to 11 nt downstream of the Cas9 cut site. The substitutions introduce synonymous mutations as well, in order to avoid a possible impact on the gene product and, hence, embryo survival and experimental outcome. The IDT CodonOpt webtool (https://eu.idtdna.com/CodonOpt) was used to determine the most optimal synonymous nucleotide changes for zebrafish. These substitutions avoid possible Cas9 cleavage of the newly edited endogenous DNA sequence, and enable correlation analysis between the mutation-to-DSB distance and HDR efficiency. The sequence and a schematic overview of all ssODN molecules are displayed in Table S8 and Fig. 1A. The ssODNs were ordered as ultramer oligonucleotides, without PAGE purification (IDT, 4 nmol) and were dissolved at a concentration of 10 µM.

### *Danio rerio* (zebrafish) maintenance and injection procedures

We adhered to the general guidelines, in agreement with EU Directive 2010/63/EU for animals, for zebrafish handling, mating, embryo collection and maintenance (Lawrence, 2007; Westerfield, 2000). Approval for this study was provided by the local committee on the Ethics of Animal Experiments (Ghent University Hospital, Ghent, Belgium; Permit Number: ECD 14/31 and ECD 17/41). Following fertilization, we micro-injected one-cell-stage AB zebrafish embryos in the cell with 1.4 nl injection mix composed of 25 pg sgRNA, 250 pg Cas9 protein (ToolGen, Cas9 wild-type nuclease protein with NLS), 2 µM ssODN template, RNase-free water and Phenol Red sodium salt indicator (Sigma Aldrich, cat. no. P4758). In specific experiments, we complemented this mixture with a chemical compound (see section ‘Compound administration’ and Table S9).

### Compound administration

The following chemical compounds were dissolved in DMSO and either supplemented to the injection mix (Table S9) or to the compound screening medium (Table S10): SCR7 (Xcessbio Biosciences Inc., cat. no. M60082-2s), KU0060648 (APExBIO, cat. no. A1769), NU7441 (Santa Cruz Biotechnology, cat. no. sc-208107), L755507 (Santa Cruz Biotechnology, cat. no. sc-204045), RS1 (Santa Cruz Biotechnology, cat. no. sc-222240). Compound screening medium consisted of 1xE3 medium supplemented with 1 mM tris/HCl (pH 7.4), 0.05 U/ml penicillin and 50 ng/ml streptomycin (Murphey and Zon, 2006). Screening medium was distributed in 96-well plates, each well containing 100 µl medium and one injected embryo. Treatment was performed for 24 h.

### Genomic DNA extraction

At 1 day post-fertilization (dpf) we categorized injected embryos as ‘dead’, ‘malformed’ or ‘normally developing’. We performed genomic DNA extraction on a pool of 20 normally developing embryos. Additionally, normally developing embryos, co-injected with CRISPR/Cas9 components targeting either *slc2a10* or *smad6a* and the corresponding symmetrical 120 nt repair template (‘NT 120 S’), were raised to adulthood. Eggs and sperm cells were collected by squeezing female and male fish at the age of 3 months according to the guidelines in ‘The Zebrafish Book’ (Westerfield, 2000). DNA extraction was carried out using the KAPA Express Extract DNA Extraction Kit (Kapa Biosystems, KK7103). Embryos, eggs or sperm were incubated at 60°C for 10 min and 95°C for 5 min in an extraction mix of 5 µl 10× Kapa Express Extract Buffer, 1 µl Express Extract Enzyme (1 U/µl) and 44 µl PCR grade water. The resulting DNA was stored at −20°C for subsequent PCR amplification.

### Sequence analysis

Using target-specific primers, the extracted DNA was singleplex PCR-amplified (Table S11). The resulting PCR products were subjected to the Nextera XT library preparation protocol (Illumina, San Diego, CA) and deep sequenced on a MiSeq instrument (Illumina) using 2×250 bp cycles (De Leeneer et al., 2015). Data analysis was performed using BATCH-GE, a Perl-based bioinformatics tool specifically designed for the assessment of NGS data resulting from target-specific genome-editing experiments (Boel et al., 2016). Adaptations to the original BATCH-GE script were made in order to be able to discriminate between perfect repair and erroneous events. The adapted analysis workflow is schematized in Fig. S5 and input parameters are shown in Table S12. The adapted script is freely available for academic use and can be downloaded from https://github.com/WouterSteyaert/BATCH-GE.git.

### Statistical analysis

Statistical analysis was carried out using SPSS statistics 24. We used the following tests: for symmetrical templates: one-way ANOVA with blocking (60 nt vs 120 nt, 60 nt vs 180 nt, 120 nt vs 180 nt, target vs non-target). For asymmetrical templates: non-parametric Kruskal–Wallis test, followed by pairwise comparison with Dunn–Bonferroni correction. For compound testing: independent samples *t*-test for normal distributed groups and the non-parametric independent samples Mann–Whitney *U*-test for non-normal distributed groups. Error bars represent standard error of mean (s.e.m.). We repeated each experiment five times to level out variability related to the injection procedure.

## Supplementary Material

Supplementary information

First Person interview
