# Supplementary material for: CRISPR/Cas9-mediated homology-directed repair by ssODNs in zebrafish induces complex mutational patterns resulting from genomic integration of repair-template fragments
Source: Dis Model Mech. 2018 Oct 18;11(10):dmm035352. doi: 10.1242/dmm.035352 (PMC6215429; doi:10.1242/dmm.035352)
Supplement: Supplementary information [file dmm-11-035352-s1.pdf]

© 2018. Published by The Company of Biologists Ltd | Disease Models & Mechanisms (2018): doi:10.1242/dmm.035352: Supplementary information

FIRST PERSON

First person – Annekatrien Boel

First Person is a series of interviews with the first authors of a selection of papers published in Disease Models & Mechanisms, helping early-career researchers promote themselves alongside their papers. Annekatrien Boel is first author on 'CRISPR/Cas9-mediated homology-directed repair by ssODNs in zebrafish induces complex mutational patterns resulting from genomic integration of repair-template fragments', published in DMM. Annekatrien is a PhD student in the lab of Paul Coucke at Ghent University, Belgium, investigating disease modeling using CRISPR/Cas9 genome editing, with a specific interest in the generation of knock-in models harboring disease-relevant point mutations.

How would you explain the main findings of your paper to non-scientific family and friends?

The study of genetic disorders relies heavily on the use of appropriate animal models of disease. Until recently, the generation of such models, harboring disease-causing mutations in the gene of interest, was quite challenging. Newly emerged genome editing techniques, of which the CRISPR/Cas9 system is most notable, have facilitated disease model generation; however, technical challenges still hamper large-scale and efficient disease model development. Many disorders are caused by specific point mutations, which are alterations in one single nucleotide pair in the DNA. The introduction of such mutations requires the administration of the components of the CRISPR/Cas9 system, alongside a repair template, which is a piece of DNA, containing the base pair alteration the researchers aim to introduce, in a process called 'homology-directed repair' (HDR). To optimize this procedure, we employed the zebrafish, which is an increasingly attractive animal to model and study human disease. Two main findings can be extracted from our study. Firstly, we observed that, in addition to the introduction of the base pair alteration of interest, complex mutational patterns of repair template fragments were introduced in the genome. Secondly, the extent to which this erroneous repair was present, was dependent on the composition of the repair template that was used.

What are the potential implications of these results for your field of research?

The finding that, and the extent to which, repair template fragments were introduced into the genome during HDR was unexpected. The technique of administrating the CRISPR/Cas9 complex along with a repair template is applied in many model organisms, as well as in human cells, and might be eventually used in therapeutic applications. Therefore, it is important to recognize the manifestation of erroneous repair during HDR, which was until now generally considered to be error-free, and to apply the appropriate techniques to analyze the efficiency and correctness of editing.

Annekatrien Boel's contact details: Center for Medical Genetics, Ghent University, C. Heymanslaan 10, 9000 Ghent, Belgium.
E-mail: Annekatrien.Boel@UGent.be

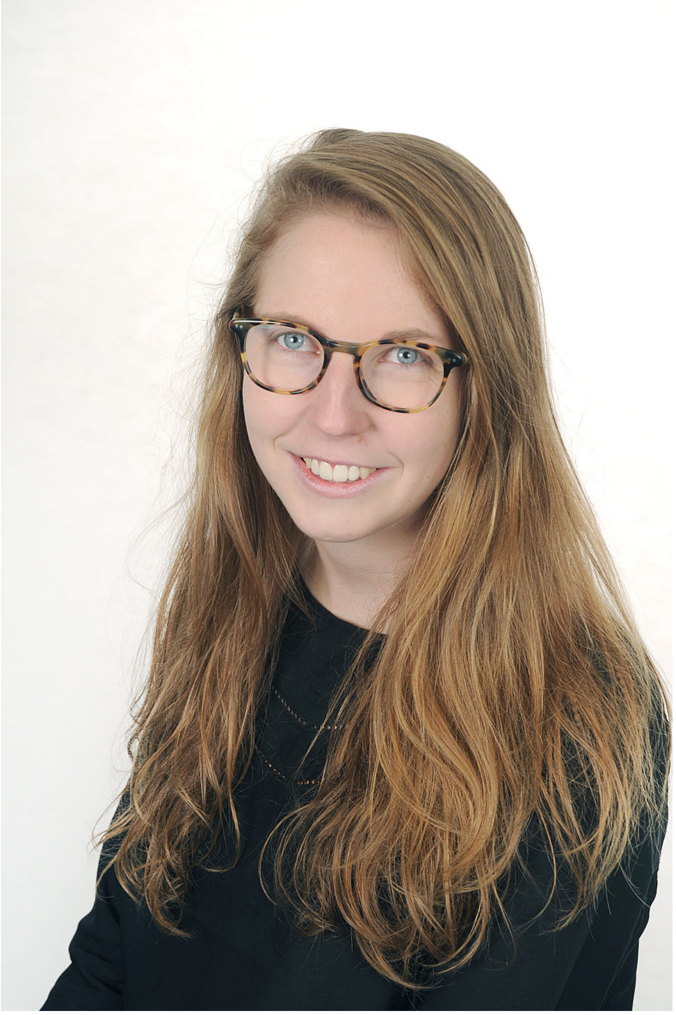

Annekatrien Boel

What are the main advantages and drawbacks of the model system you have used as it relates to the disease you are investigating?

> "These characteristics make zebrafish the ideal *in vivo* disease model to conduct large-scale genome editing studies."

The main strength of zebrafish, in comparison to pre-eminently used model systems, such as rodents, is its high-throughput capability, displaying a high fecundity, *ex vivo* fertilization, and a rapid and *ex utero* development. These characteristics make zebrafish the ideal *in vivo* disease model to conduct large-scale genome editing studies. One of its main advantages can also turn into a disadvantage: a rapid development means that zebrafish undergo fast consecutive cell division cycles, resulting in highly mosaic F0 animals harboring a multitude of different mutations.

What has surprised you the most while conducting your research?
In one specific experiment of our study, we investigated the influence of five chemical compounds previously shown to improve HDR rates in cellular systems. Surprisingly, at four different target sites in the zebrafish genome, none of the administered compounds had a notable influence on HDR rates. While this outcome was rather disappointing, we believed it was still useful to include these results in our manuscript. Publishing experimental set-ups that do not work might save other research groups precious time and effort.

> "Publishing experimental set-ups that do not work might save other research groups precious time and effort."

Describe what you think is the most significant challenge impacting your research at this time and how will this be addressed over the next 10 years?
While the emergence of CRIPSR/Cas9 genome editing is one of the most exciting developments of recent years in the field of genetics, the way to its implementation in medicine is still long. Findings like ours absolutely do not undermine the power of this technique. They do, however, contribute to the establishment of CRISPR/Cas9 as a safe technique by pinpointing the challenges that come along with it. In coming years, researchers will continue to do so and each possible risk regarding specificity or accuracy should be addressed appropriately in future applications. The ever-decreasing cost of next-generation sequencing techniques will be absolutely beneficial in this regard.

What's next for you?
After completion of my PhD, I will start work as a postdoctoral fellow, supporting different research groups that aim to implement the CRISPR/Cas9 genome editing technique in their research projects.

Reference
Boel, A., De Saffel, H., Steyaert, W., Callewaert, B., De Paepe, A., Coucke, P. J. and Willaert, A. (2018). CRISPR/Cas9-mediated homology-directed repair by ssODNs in zebrafish induces complex mutational patterns resulting from genomic integration of repair-template fragments. *Dis. Model. Mech.* **11**: dmm035352.
